# Novel Approaches to an Integrated Route for Trisomy 21 Evaluation

**DOI:** 10.3390/biom11091328

**Published:** 2021-09-08

**Authors:** Angelika Buczyńska, Iwona Sidorkiewicz, Anna Trochimiuk, Sławomir Ławicki, Adam Jacek Krętowski, Monika Zbucka-Krętowska

**Affiliations:** 1 Clinical Research Centre, Medical University of Bialystok, 15-276 Bialystok, Poland; iwona.sidorkiewicz@umb.edu.pl (I.S.); adamkretowski@wp.pl (A.J.K.); 2 Department of Endocrinology, Diabetology and Internal Medicine, Medical University of Bialystok, 15-276 Bialystok, Poland; anna.trochimiuk@umb.edu.pl; 3 Department of Population Medicine and Civilization Diseases Prevention, Medical University of Bialystok, 15-276 Bialystok, Poland; slawicki@umb.edu.pl; 4 Department of Gynecological Endocrinology and Adolescent Gynecology, Medical University of Bialystok, 15-276 Bialystok, Poland

**Keywords:** trisomy 21, metabolomics, genomics, prenatal screening

## Abstract

Trisomy 21 (T21) is one of the most commonly occurring genetic disorders, caused by the partial or complete triplication of chromosome 21. Despite the significant progress in the diagnostic tools applied for prenatal screening, commonly used methods are still imprecise and involve invasive diagnostic procedures that are related to a maternal risk of miscarriage. In this case, novel prenatal biomarkers are still being evaluated using highly specialized techniques, which could increase the diagnostic usefulness of biochemical prenatal screening for T21. From the other hand, the T21′s pathogenesis, caused by the improper division of genetic material, disrupting many metabolic pathways, could be further evaluated with the use of omics methods, which could result in bringing relevant insights for the evaluation of potential medical targets. Accordingly, a literature search was undertaken to collect novel information about prenatal screening for Down syndrome with the use of advanced technology, with a particular emphasis on the evaluation of novel screening biomarkers and the discovery of potential medical targets. These meta-analyses are focused on novel approaches designed with the use of omics techniques, representing the most rapidly developing and promising field in research today. Considering the limitations and progress of these methods, the use of omics techniques in evaluating T21 pathogenesis could bring beneficial results in prenatal screening, simultaneously uncovering novel potential medical targets.

## 1. Introduction

Trisomy 21 (T21), also known as Down syndrome, is one of the most frequently occurring chromosomal aberrations, appearing in 1 in 319 to 1 in 1000 live births [1]. The most frequently diagnosed duplication of chromosome 21 as a result of the abnormal nondisjunction of chromosomes occurs in an estimated 95% of cases, and the remaining 5% are associated with translocation and somatic mosaicism [1,2,3]. T21 patients struggle with physical and mental disabilities and many others comorbidities, such as heart defects, thyroid disease, leukemia, cancers, Alzheimer’s disease, and others [1,3,4,5]. The clinical manifestation of an additional chromosome 21 determines the well-recognized phenotype, which includes an altered facial appearance (flatness of the bridge of the nose, midfacial hypoplasia, and a tendency to protrude the tongue) and musculoskeletal features (inflammatory arthritis, scoliosis, and patellar instability) [1,3,6].

The prenatal screening for T21 is currently based on noninvasive methods, which enable the estimation of the risk of its occurrence, and invasive techniques, mainly used to verify the presence of chromosomal aberrations. Serum screening and ultrasound are used to identify women whose pregnancies are at a high risk of chromosomal abnormalities. Positive screening results can lead to the need to undergo invasive procedures, such as amniocentesis or chronic villus sampling (CVS), where the 21-trisomic karyotype can be confirmed. CVS involves the aspiration of placental tissue, and amniocentesis involves the collection of amniotic fluid. Although invasive techniques are characterized by high diagnostic specificity, they are also associated with a 1% risk of miscarriage [6,7]. On the other hand, material collected using invasive techniques, such as amniotic fluid, is still useful for research [8]. The breakthrough moment in prenatal diagnosis was the development of noninvasive cell-free fetal DNA (cffDNA) evaluation; however, its high cost has limited the introduction of this test into routine management. There has been significant development of diagnostic tools for prenatal diagnosis, but the number of patients undergoing invasive tests remains constant [7,8]. Therefore, it is still important to find a cost-effective and noninvasive screening method for biomarker discovery characterized by high sensitivity and specificity, which would provide certain benefits, subsequently leading to the evaluation of novel medical targets. In this case, the application of novel biochemical screening markers, determined using specific novel techniques, may lead to a reduction in unnecessary invasive procedures [9].

Clearly, there is still a need to evaluate the insufficiencies in metabolic pathways involved in the patomechanism of T21. Bioinformatics has enabled comprehensive multi-omics and clinical data integration for insightful interpretation. In this review, we outline considerations of omics methods applied to experimental design and general frameworks for the integration of omics data in T21 research, along with analytic strategies, and speculate about future multi-omics approaches. Information about T21 prenatal screening received with use of advanced technology, with a particular emphasis on the evaluation of novel screening biomarkers and the discovery of potential novel medical targets, was collected. We hope that this study will also provide novel insights to improve the management of complications related to the genetic, metabolomic, and proteomic disturbances observed in T21 development, while also providing possible insights into the role of prenatal screening.

## 2. Materials and Methods

**Figure 1:**
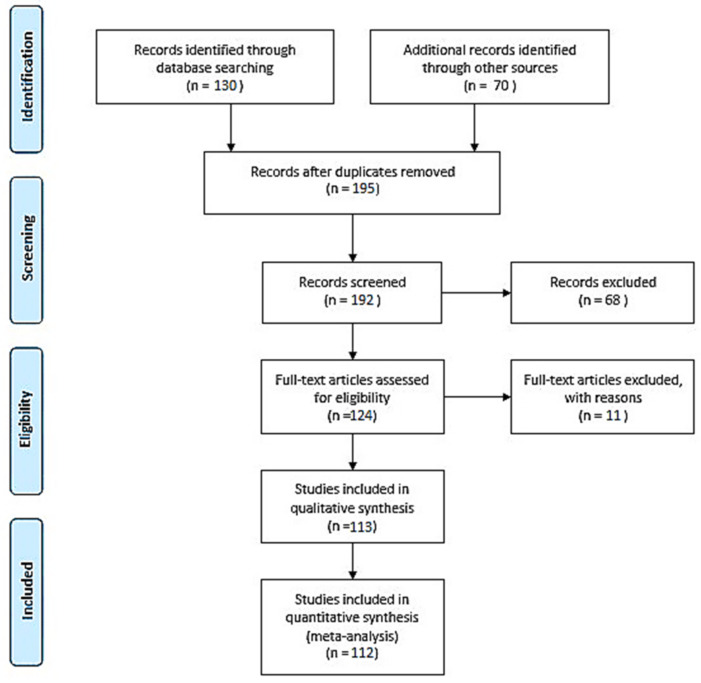
PRISMA flow diagram of meta-analysis process performed during research [13].

The literature evaluation was conducted using the PubMed database following the PRISMA and EQUATOR network guidelines [10,11,12,13]. We considered medical papers published in 2000–2021. The papers were independently selected and reviewed. The impact factors of the journals used in this study ranged from 1.14 to 70.67. Articles with irrelevant conclusion statements or inappropriate study methods, inadequate reporting, or dissemination of incomplete reports were excluded from the study (Figure 1).

To assess the diagnostic tools for prenatal screening markers, the receiver operating characteristic (ROC) curve and the area under the ROC curve (AUC) were taken into consideration [14].

## 3. Current Recommendation for Down Syndrome Screening

The screening for T21 is currently based on noninvasive methods using serum biomarkers and ultrasound examination. First-trimester aneuploidy screening is performed during the 11th to 13th weeks of gestation and includes the measurement of nuchal translucency by ultrasound and maternal serum-free beta-human chorionic gonadotrophin (βhCG) and pregnancy-associated plasma protein A (PAPP-A) [15]. 

Lately, the second-trimester screening for T21 has been primarily assigned to lower levels of maternal serum alpha fetoprotein (MSAFP) and unconjugated estriol with elevated βhCG and inhibin a concentrations. An increased concentration of MSAFP was associated with open spina bifida in the fetus [16]. The most reliable serum biomarkers—βhCG, alpha-fetoprotein (AFP), unconjugated estriol (E3), and PAPP-A—were associated with 5–10% rates of false positives (Table 1) [2,7,17,18,19]. 

In the last decade, the first-trimester prenatal screening replaced the performance of second-trimester measurements. It was proved that first-trimester prenatal screening biochemical tests, when combined with the ultrasound marker of fetal NT thickness, are more reliable, thus detecting more than 90% of the cases [15,25].

Due to the rapid development of promising untargeted omics evaluations using advanced technology for the comprehensive comparative analysis of genomes, knowledge of the metabolome and proteasome could enhance the diagnostic use of prenatal screening. These methods are characterized by high sensitivity, specificity, and reproducibility. Moreover, these studies enable the dissemination of knowledge about the disturbances of metabolic pathways involved in T21 development, which could reveal unanticipated metabolic perturbations and lead to the discovery of novel medical targets. The detection of fetal cells and fetal DNA circulating in maternal blood has increased the role of prenatal screening [26,27]. The progressive integration of different omics methods in T21 pathogenesis could elucidate potential causative changes that lead to this disease or determine the treatment targets that could be studied in further clinical trials.

## 4. Prenatal Genetic Diagnosis of Down Syndrome

Modern genetics started with research by the Augustinian friar Gregor Johann Mendel, published in 1866, when the theory of Mendelian inheritance was established [28]. The next generation and the rapid development of science led to the discovery of DNA as the structure of chromosomes in 1950 [29]. In 2003, the successful completion of the Human Genome Project, with 99% of the genome sequenced at a 99.99% accuracy, was a breakthrough in genomic development. In the past few decades, many biologists have focused on large-scale genetics projects based on clinical diagnosis and medical intervention possibilities for many diseases [30]. The basic method used to determine trisomy 21 is amniotic fluid chromosome analysis [31]. Amniocentesis is usually performed between the 15th and 18th gestational weeks. Although invasive techniques are associated with a risk of miscarriage, the removal of amniotic fluid itself causes no harm to the developing fetus and is optimal for obtaining fetal cells for culture. Metaphase chromosome analysis or chromosomal microarray analysis (CMA) is almost always performed on amniotic fluid samples [32].

In order to avoid invasive prenatal procedures, there is a need for new T21 biomarkers that have high sensitivity and specificity. Thus, genetic tests could be implemented for noninvasive T21 screening panels. Since the presence of fetal DNA in maternal plasma and serum was established by Lo et al. in 1997 using genome sequencing techniques, rapid progress has been observed in prenatal genetic testing [23]. Gene identification is important for understanding the pathophysiology of diseases and improving diagnosis, prevention, and treatment. The complete sequencing of chromosome 21 provided a basis for the identification of candidate genes for T21 phenotype manifestations. The mechanisms by which an extra copy of chromosome 21 produces the phenotypes of T21 are complex. Next-generation sequencing (NGS) technology is not limited to gene chip technology, so the identification of novel genes is less time-consuming and expensive. Moreover, post-data analysis has been improved by the establishment of huge public data repositories.

NGS technology facilitates a high detection rate and a low percentage of false positive T21 results [24]. There are several genes located on chromosome 21 associated with T21 phenotypes. The genes that have been implicated in T21 development include Cu/Zn superoxide dismutase (SOD1), amyloid precursor protein (APP), Ets-2 transcription factors, Down syndrome critical region 1 (DSCR1) stress-inducible factor, beta-site APP cleaving enzyme (BACE), and S100 [33,34]. The Down syndrome critical region (DSCR) is a chromosome 21 segment purported to contain genes responsible for many features of T21 [35]. The DSCR hypothesis predicts that genes in this region are sufficient to produce T21 phenotypes. Studies should evaluate the association of DSCR with T21 diagnosis. The involvement of other genes can be elucidated with advances in omics methods. Sequencing methods are characterized by higher accuracy than chromosome analysis and can detect gene variation effectively. Moreover, this type of prenatal screening is not dependent on gestational age [25]. Compared with traditional first-generation sequencing technology, the sequencing of the human genome initiated the discovery of the T21 patomechanism, leading to a growing understanding of the genetic determinants of this disease, resulting in noninvasive prenatal screening (NIPT) [24].

Rapid improvements in genetic technologies led to the evolution of the prenatal screening of cffDNA, which is more sensitive and specific than biochemical screening methods. From early pregnancy, cffDNA is present in maternal blood, the majority of which originates from the mother herself, but with relevant fetal components contributing approximately 10–20% of the total. Most cffDNA is derived from villous cells; its concentration increases with increasing gestational age, and it is rapidly cleared from the maternal circulation within hours of delivery, making it pregnancy-specific [36]. This measurement is highly specific with regard to representing the entire fetal genotype [28]. The rapid development of massively parallel sequencing (MPS) technology made it feasible to use maternal plasma cffDNA to detect trisomy 21. However, relying on complex and expensive MPS techniques hinders the use of cffDNA as a common screening procedure [37]. With noninvasive tests of maternal blood (fetal and maternal) circulating free DNA (cfDNA) and cffDNA (originating from placenta), results that are discordant with the fetal karyotype can arise from the detection of maternal chromosomal rearrangements or mosaicism, maternal malignancy, or confined placental mosaicism. However, false negatives can occur in cases of decreased concentrations or inconsistent laboratory techniques. Moreover, NIPT is not considered as a diagnostic tool in less economically developed countries, and the confirmation of positive results by invasive testing is still required [29]. Various factors affect the accuracy of ccfDNA results, including confined placental mosaicism, the contribution of maternal DNA, and technical or statistical issues [38].

Currently, the NIPT field is dominated by the cffDNA approach. However, cell-based NIPT (cbNIPT) has been proposed as a superior alternative to overcome the challenges associated with cffDNA [39]. Trophoblasts, granulocytes, lymphocytes, stem cells, and nucleated red blood cells (nRBC) have been identified in maternal blood as a source of cell-based DNA (cbDNA) [40,41,42]. Intact fetal cells harvested from the maternal circulation represent the uncontaminated fetal DNA which enable to avoid the issues associated with using fragmented cffDNA. A study by Vossaert et al. has demonstrated no significant correlation between maternal age, body mass index, and trophoblast yield of single circulating trophoblast testing, which proves the advantage over cffDNA testing [43].

One of the most limiting factors in cbDNA procedure is to isolate the rare fetal cell from maternal circulation. Fingerprinting by short tandem repeat analysis, fetal cell enrichment, and staining, cell sorting based on physical characteristics, antigens, and proteins have been proposed as useful methods to obtain information regarding the cellular origin [44]. Recently, automated fetal nRBC and extravillous trophoblast capture systems have been validated in the genetic diagnosis [45,46]. Nevertheless, insufficient clinical trials able to provide evidence demonstrating a robustness of cbDNA and its diagnostic value in fetal aneuploidy diagnosis still restrain its clinical implementation [47].

Altered RNA expression is observable for many but not all of the genes mapped on chromosome 21 and for a larger number of genes located on other chromosomes. Epigenetics refers to the regulation of gene expression through microRNA synthesis, DNA methylation, and histone modification processes. Data in the literature suggest that DNA methylation, as a mechanism regulating gene expression, plays an important role in the pathogenesis of T21 [48,49]. DNA methylation is a chemical modification of the fifth carbon of a cytosine base to form 5-methylcytosine (5-mc) catalyzed by DNA methyltransferases. Studying the baseline epigenetic effects on chromosome 21 is a useful approach for evaluating novel therapies. Studies focused on the epigenome-wide evaluation of T21 have identified 1052 differentially methylated regions associated with this disease, including significant hypermethylation regions of RUNX family transcription factor 1 (RUNX1) and *Fli-1* proto-oncogene (FLI1), the main regulators of hematopoiesis [48]. Furthermore, it was proved that reduced neuron-restrictive silencer factor/RE1-silencing transcription factor (NRSF/REST) expression with the simultaneous upregulation of DYRK1A (mapped on chromosome 21q22.13) and protocadherin gamma cluster (PCDHG) gene expression observed during early T21 development may contribute to insufficient neural circuit formation in the developing brain. The upregulation of DNMT3L (on chromosome 21q22.4) could additionally lead to de novo methylation during neurodevelopment, resulting in DNMT3A and DNMT3B downregulation in the brains of T21 fetuses [50]. The epigenetic signature of T21 is mainly enriched in genes responsible for hematopoiesis, morphogenesis, and development, and the regulation of the chromatin structure in neurons [51]. This observation may provide useful novel biomarkers for T21 brain development and potential novel medical targets for prenatal therapeutic interventions.

Analyses of novel markers in T21 screening have mainly focused on noncoding nucleic acids such as microRNA (miRNA). A miRNA is a single, non-coding RNA molecule that can be obtained from the maternal compartment as a useful diagnostic tool to identify fetal T21 occurrence [22,28]. MiRNAs can affect protein expression by interfering with RNA translation or promoting mRNA degradation [32,33]. Combined with DNA methylation, miRNA provides a means to evaluate changes in gene activation and expression, and to understand the impact on gene clusters that affect particular pathways [34,35]. Data in the literature prove that T21 is associated with multiple patterns of deregulation in maternal plasma miRNA expression, such as that of let-7c, miRNA-99a, miRNA-125b, miRNA-155, miRNA-802, miRNA-3118, miRNA-3156, miRNA-3196, miRNA-3648, miRNA-3687, miRNA-4327, miRNA-4759, and mir-99a [32,34,35,36]. This differential expression is related to the occurrence of neuropathology, leukemia, hematopoiesis, congenital heart defects, and autism during T21 development [32,33,37,38]. Moreover, altered expression of miR-1973, miR-3196, and miR-138 related to T21 comorbidities has also been reported [38,39,40]. Deregulated expression of miR-138-5b and miRNA-155 has a significant impact on hippocampal tissues from T21 fetuses, and the downregulation of this target may be involved in intellectual disability and neurological deficiency [35,38,41]. Zbucka-Krętowska et al. revealed 13 miRNAs differentially expressed—six miRNAs upregulated (hsa-miR-15a, hsa-let-7d, hsa-miR-142, hsa-miR-23a, hsa-miR-199 and hsa-miR-191) and seven downregulated (hsa-miR-1290, hsa-miR-1915, hsa-miR30e, hsa-miR-1260, hsa-miR-483, hsa-miR-548 and hsa-miR-590)—in maternal plasma obtained from T21 pregnancies, which were considered to make up a potential noninvasive second-trimester prenatal screening panel [35]. The study was conducted on 12 patients with fetal DS and 12 patients with uncomplicated pregnancies considered as the control group, using NanoString technology, with the determination of the expression levels of 800 miRNAs. Prenatal biomarkers play an essential role in early diagnosis, prediction and clinical management [28]. Since the pathophysiology of T21 is extremely complicated, determining the disturbed metabolic pathways is highly recommended. It can be hypothesized that assessing the trisomy 21-induced overexpression of chromosome 21-derived miRNAs will become the standard of T21 diagnostics in the future [52]. Moreover, a study performed by Erturk et al. showed that the suggested variation in miR-155 expression commonly observed with miR-802 assessed in T21 tissues was associated with immunological complications, in particular, with the upregulation of CD4+ T cells. For this study, 56 patients underwent invasive prenatal testing, 23 of which were carrying fetuses affected by Down syndrome, and 33 control cases were included for comparison. All the biological material was collected during the 17th and 18th weeks of gestation, and the miRNA expression levels were measured using real-time RT-PCR. In this case, differentiation into Th-1 lymphocytes, leading to a reduced number of embryonic B cells and extra-follicular B cells, was observed. The deregulation of microRNA expression may be the reason for the reduced synthesis of high-affinity IgG antibodies observed in T21 patients [30,34]. These results could also be analyzed using novel medical target approaches.

Undeniably, genomics is still a new field in science. As mentioned, cffDNA has had a significant influence on the evolution of prenatal T21 screening, and the promise of miRNA is under constant examination. On the other hand, genetic tests are characterized by limitations in sensitivity because only a subset of causative mutations can be identified simultaneously. Furthermore, the sensitivity of the test may be influenced by ethnicity. The detection of global changes in miRNA expression and the subsequent interpretation of such data may additionally be dependent on the specific platform used [53]. Novel genetic technologies could enhance the diagnostic sensitivity. Genomic instability, aneuploidy and other polymorphism-based variations that originate in the female germline and contribute to developmental defects during T21 development can be determined through investigations based on sequencing and epigenetic techniques. However, the high cost and complex nature of post-data metanalyses currently limit the worldwide implementation of this procedure, especially in underdeveloped and moderately developed countries [52,54]. Molecular genetic techniques augment chromosome analysis, broadening the range of identifiable genetic abnormalities, and may accelerate the clinical management of patients [55].

## 5. Metabolomic Profiles as Down Syndrome Markers

Metabolomic methods are mainly based on nuclear magnetic resonance (NMR) spectroscopy and mass spectrometry (MS) [56]. NMR and MS are powerful analytical techniques used to quantify unknown/known biological materials, identify unknown compounds in samples and elucidate the structure and chemical properties of different molecules, with the subsequent evaluation of concentrations. NMR is characterized by the chemical shift of protons (H-1 NMR) or carbon (13C-NMR) atoms. The shift depends on the range of atoms in the subject atom’s vicinity. A mass spectrometer generates multiple ions from the sample under investigation, followed by separation based on their specific mass-to-charge ratio (*m*/*z*). Thus, records of the relative abundance of each ion type are established [57]. In comparison with genomics, metabolomics techniques can be used to analyze pleiotropic molecular metabolites obtained from biological compartments, the quantitative determination of which could be considered in screening novel biochemical and, in the future, evaluating treatment follow-up markers [58]. The results obtained from maternal plasma and amniotic fluid evaluation are reliable and have been validated for the discovery of novel T21 screening biomarkers [58,59,60].

Following a study performed by Bahado-Singh et al. using NMR-based metabolomics, 11 maternal serum novel metabolites (2-hydroxybutyrat, 3-hydroxybutyrate, 2-hydroxyisovalerate, acetamide, acetone, carnitine, dimethylamine, lactate, methionine, pyruvate and L-methylhistidine) were revealed as being significantly different between T21 and euploid pregnancies, and, more importantly, three of the examined molecules (3-hydroxybutyrate, 3-hydroxyisovalerate and 2-hydroxybutyrate) were reported to have increased concentrations during the first trimester of pregnancy. These metabolites are produced as a result of complementary mechanisms, and are involved in myelination and in the prevention of increased levels of oxidative stress, which are confirmed in T21 pathogenesis. Furthermore, 3-hydroxybutyrate is a ketone, which is an important substrate for phospholipid and sphingolipid synthesis. Accordingly, both phospholipids and sphingolipids are required for neuronal transition processes and myelination.

Regarding sphingolipid pathways, Charkiewicz et al. proved the second-trimester screening utility of measuring selected sphingolipids in the maternal plasma and amniotic fluid [61]. A significant increase in the levels of two ceramides, C22-Cer (AUC = 0.814) and C24:1-Cer (AUC = 0.729), in the T21 pregnancies was observed. On the other hand, decreases in the concentrations of seven ceramides were reported: C16-Cer (AUC = 0.857), C18-Cer (AUC = 0.968), C18:1-C (AUC = 0.897), C20-Cer (AUC = 0.960), C22-Cer (AUC = 0.873), C24:1-Cer (AUC = 0.905), and C24-Cer (AUC = 0.802) [61]. The study was conducted on samples from 10 pregnancies with confirmed Down syndrome between the 15th and 18th gestational weeks using ultra-high performance liquid chromatography coupled with triple quadrupole mass spectrometry (UHPLC/MS/MS).

In another extended study, Parfieniuk et al. performed plasma metabolomics using liquid chromatography–mass spectrometry (LC-MS), using samples obtained from 12 pregnancies with confirmed fetal T21, and 15 pregnant women with euploid fetus consisted as a control group, being between the 15th and 18th gestational weeks, and reported a significant decrease in five maternal metabolites: butyryl-l-carnitine, palmitic amide, linoleamide, oleamide, and piperine. The combination of linoleamide and piperine was reported to have higher sensitivity and specificity in the screening of T21 aberrations. Palmitic amide, linoleamide, and oleamide are also known as fatty acid amides (FAAs) and have been described as molecules able to block gap junction communication in glial cells, with a relevant impact on memory processes, the stimulation of Ca^2+^ release, and the activation of serotonin and endocannabinoid receptors [59]. Piperine, an exogenous alkaloid, is characterized by anti-inflammatory, antioxidant, antipyretic, antidiarrheal, and gastro- and neuroprotective properties. Therefore, the observed decreased piperine level could be another reliable biomarker of insufficient nervous system development in T21 fetuses (Table 2) [62].

A study conducted by Nemutlu et al. used a metabolomic platform, gas chromatography–mass spectrometry (GC-MS), and liquid chromatography–quadrupole time-of-flight mass spectrometry (LC-qTOF-MS) to find possible metabolites differentiating between healthy/normal and T21 pregnancies that could confidently be used for T21 screening. This study noted significant alterations in the concentrations of l-threonic acid, beta-alanine, oxalic acid, creatinine, alpha-tocopherol, cholesterol, uracil, and 2-piperidone, associated with an increased risk of T21 occurrence [64]. All of these metabolites previously showed vital roles in fetal development, such as beta-alanine (an antioxidant), which is the building block of carnosine and has been associated with extended muscular endurance in pregnancy. Alpha-tocopherol, a naturally occurring form of vitamin E (antioxidant), participates in lipid metabolism and regulates oxidative stress status, which is essential for proper fetal (brain) development. Furthermore, uracil has neuroprotective properties as a substrate of uracil-DNA glycosylase and uridine phosphorylase enzymes. These enzymes also eliminate mediators of oxidative stress, providing protection against brain neurodegeneration. The decreased plasma levels of uracil observed in T21 pregnancies could potentially be associated with fetal neurodegeneration and increased oxidative stress and lipid peroxidation [64].

In summary, metabolomics has been considered as a powerful tool for identifying novel T21 screening biomarkers. However, one should bear in mind the relevant differences in the patients’ genotypes, medical histories, disease development, ethnicities, and diets and ages, which might affect the metabolome and directly influence the obtained results. Additionally, as metabolomics evaluation is usually based on different platforms, analytical protocols with different sample preparation methods and data analysis techniques may also contribute to controversial and divergent outcomes [65,66]. Furthermore, metabolomics studies require highly trained personnel and huge financial investment, which may not be feasible at the clinical level for the purpose of T21 screening. However, these methods are widely used in the determination of disturbed metabolic pathways, constituting a foundation for research in the development of novel screening markers, and are more valuable in evaluating possible medical targets. It is still impossible to designate a uniform T21 treatment; however, efforts are ongoing to devise personalized therapy for T21 treatment using state-of-the-art omics (genomic, proteomic, and metabolomic) approaches. Last but not least, metabolomics is a novel, useful tool for determining insufficiencies in metabolic pathways in T21 and T21 pregnancies, with potential for the evaluation of novel medical targets. However, there is still a need to find cost-effective techniques for validation.

## 6. Proteomics and Down Syndrome Screening

The proteome describes the protein component expressed in cells and tissues. By using proteomic techniques, isoforms and protein post-translational variants can also be evaluated. In addition, post-translational modifications such as phosphorylation, ubiquitination (which modulates protein activity and mediates signal transduction), and proteolytic cleavage can be determined. Current proteomics use MS with LC-MS-MS and matrix-assisted laser desorption/ionization (MALDI) equipment [67]. The MALDI method is based on an ionization technique that uses a laser energy-absorbing matrix to create ions from large molecules with minimal fragmentation.

Proteomics could also enable the detection of novel and more affordable T21 biomarkers [68,69]. Accordingly, Charkiewicz et al. suggested that imbalance in the level of circulating proteins in maternal blood can stimulate an immune response producing autoantibodies. In this study, 190 amniocenteses were performed, and 10 patients with confirmed fetal Down syndrome (15th–18th weeks of gestation) were found. Statistical analysis of the expression of 9000 autoantibodies in T21 pregnancies, revealed using a protein microarray, which allows for the simultaneous determination of 9000 proteins per sample, showed that the expression of 213 autoantibodies was significantly different when compared with that in euploid pregnancies. Moreover, this panel could potentially be used in prenatal T21 screening, based on the specification of the predictive value (specificity and sensitivity) equal to 100%, 0% classification errors, and 0% cross-validation errors [70].

Following the evaluation of disturbed immune response, Laudanski et al. indicated that chemokine measurement could also be relevant in prenatal T21 screening. Based on a protein microarray, that study reported that seven women with fetal DS in the 15th–18th weeks of gestation had increased plasma concentrations of one chemokine, CXCL7 (NAP-2), and decreased plasma concentrations of four chemokines, hemofiltrate CC chemokine 4 (HCC-4), interleukin 28A (IL-28A), interleukin 31 (IL-31), and monocyte chemotactic protein 2 (MCP-2). The MCP-2 measurement was characterized by the highest diagnostic value, based on AUC = 0.830 [71]. Research performed by Zbucka-Kretowska et al. demonstrated significant increases in the T21 maternal plasma concentrations of four angiogenic factors (transforming growth factor beta 1 (TGFb1), angiostatin, chemokine (C-C motif) ligand 1 (I-309), transforming growth factor beta 3 (TGFb3), and vascular endothelial growth factor D (VEGF-D)), and one antiangiogenic (angiostatin), and decreases in the concentrations of 14 angiogenic factors (leptin, angiopoietin 1 (ANG-1), angiostatin, epidermal growth factor (EGF), interleukin 1-beta (IL-1b), interleukin 4 (IL-4), interleukin 12p40 (IL-12p40), MCP-2, matrix metalloproteinase-1 (MMP-1), matrix metalloproteinase-9 (MMP-9), platelet endothelial cell adhesion molecule 1 (PECAM-1), transforming growth factor alpha (TGF alpha), vascular endothelial growth factor 2 (VEGFR2), and vascular endothelial growth factor 3 (VEGFR3)). The study used protein microarrays, which enable the simultaneous determination of 60 angiogenic factors per sample [72]. It was conducted on 20 patients with T21 fetuses and a control group of 28 healthy patients with uncomplicated pregnancies in women who delivered healthy newborns at term. The biological material was collected during the 15th–18th weeks of gestation. Based on bioinformatic analysis, these disturbances were associated with tissue remodeling, bone formation during embryogenesis, and the insufficient immune system activity observed during T21 fetus development [72,73].

Proteomics can also be used to determine disturbed metabolic pathways. Many studies have reported the overexpression of several plasma proteins as a result of trisomy 21 development [74,75,76,77]. In evaluating duplicated chromosome 21 genes, several antiangiogenic factor genes were mapped. These gene abnormalities were the basis for subsequent research in the field of disturbed protein expression, resulting in specific changes in T21 pregnancy proteinograms. Several proteins have been shown to be differentially expressed in T21 maternal serum [68,78]. A study by Kolialexi et al., using Western blotting, found that the plasma transthyretin (THY), ceruloplasmin (CERU), afamin (AFAM), alpha-1-microglobulin (AMBP), apolipoprotein E (APOE), serum amyloid P-component (SAMP), and histidine-rich glycoprotein (HRG) concentrations were upregulated, with a simultaneous decreased concentration of clusterin (CLUS) [79]. In this study, plasma obtained from eight women carrying DS fetuses and twelve with non-DS fetuses was analyzed using two-dimensional gel electrophoresis (2-DE) and matrix-assisted laser desorption/ionization time-of-flight mass spectrometry (MALDI-TOF-MS). Three proteins (AFAM, CERU, and TTHY) are involved in carrying factors, such as fat-soluble vitamin E, copper, a thyroid hormone, thyroxine (T4), and retinol-binding protein bound to retinol. These results were also associated with poor pregnancy outcomes [80]. These proteins are necessary for proper hormone synthesis, antioxidant defense, and cell development. CLUS, an acute phase protein, is involved in diseases related to oxidative stress [81]. In this case, the disturbed protein profile observed in the maternal compartment resulting from T21 pregnancy could be related to the many comorbidities observed in T21 fetuses [79,82,83].

Sui et al. reported increased levels of seven proteins (oxoglutarate dehydrogenase L (OGDHL), serum amyloid P component (SAP), ApoE, nucleosome assembly protein 1-like 1 (NAP1L1), thymosin beta 10 (Tβ10), complement factor B, and endoplasmic reticulum oxidoreductase 1 alpha (ERO1L)) in maternal plasma and umbilical cord blood obtained from T21 pregnancies [83]. The study was conducted on maternal peripheral blood (eight with fetal DS and eight with normal fetuses) using Western blotting. OGDHL is a functionally active isoenzyme of oxoglutarate dehydrogenase (OGDH) present in brain tissue, the main malfunction of which is related to neurodegeneration [83]. SAP can interfere with lipoprotein metabolism by activating and regulating amyloid formation [83]. NAP1L1 has a prominent role in the early development of cardiac or stem cells that differentiate into myocardial cells [83]. Tβ10 is related to cell proliferation, cell morphology, cell migration, and endocytosis and participates in cytoskeleton assembly. It can be hypothesized that the overexpression of SAP, NAP1L1, and Tβ10 proteins is associated with cognitive impairment in T21 individuals and the early development of Alzheimer’s disease (AD) observed in early-stage T21 development [83,84].

In summary, the application of proteomic technologies to the evaluation of biological compartments creates novel possibilities for elucidating the patomechanism and discovering novel drug targets and early disease markers. At the same time, proteomic results showing how sets of proteins interact with environmental factors are constantly changing [85]. Furthermore, the concentrations of many proteins depend on their locations in biological compartments and the phase of the cell cycle, which can also be interrupted by many diseases [68,69]. However, the extensive software required for utilizing proteomic data and the need for highly proficient technicians substantially increase the cost. Moreover, quality control has not yet been developed, so the clinical requirements are not met [86]. It should be emphasized that proteomics has already contributed to significant progress being made in determining insufficient biological pathways in T21 aneuploidy.

## 7. Single-Protein Determination 

Single proteins can be measured using different methods, but the enzyme-linked immunosorbent assay (ELISA) is the one most often used. This is a plate-based method used in a wide range of diagnostic laboratories around the world, designed for the sensitive and quantified measurement of soluble substances such as peptides, proteins, antibodies, steroids, and glycoproteins [87]. ELISA can be used in many settings, including the clinical diagnosis of human diseases. Based on its cost-effectiveness and uncomplicated protocols, not involving complicated sample pre-treatment, this method is an important part of medical care and scientific research [88]. This method could also be a useful tool for meeting the challenge of introducing results obtained with omics-based methods into daily routine diagnostics while also validating procedures. Unfortunately, it cannot be ignored that traditional ELISA is time-consuming and imprecise, on account of the evaluation of one variable (substance), compared with metabolomics or proteomics, in which the entire metabolome and proteome can be studied simultaneously. In this case, the multiplex ELISA-based method could be a corresponding modification to meet these requirements [89]. However, single biomarkers are not likely able to serve as the best diagnostic or prognostic markers for T21 due to their limited discriminatory power. On the other hand, biomarker panels comprising multiple measured analytes provide high sensitivity and specificity for distinguishing T21 from euploid pregnancies [90].

ELISA is still widely used in research on improving the utility of recommended prenatal screening. A study performed by Chambers et al. revealed that the additional assessment of βhCG with its cognate receptor (hCG-sLHCGR) increased the diagnostic usefulness of single-protein prenatal measurements. A comparison of the assessed methods for prenatal screening with received AUC values is presented in Table 3.

To date, several proteins have been determined by novel T21 prenatal screening applications. It is suggested that proteins related to lipid metabolism may be of great importance in the T21 patomechanism, and therefore in diagnostics. Furthermore, the relationship between maternal ApoE and fetal T21 occurrence was previously suggested through polymorphism evaluation [91]. Studies suggest that an increased maternal frequency of the APOE4 allele should be considered a risk factor of T21 occurrence [92,93]. Moreover, APOE ε4 is associated with a worse prognosis in early development for individuals with DS [94]. Following the evaluation of ApoE polymorphism, the screening utility of ApoE measurement in second-trimester T21 screening was analyzed. Considering this preliminary study, the plasma concentration of ApoE was significantly higher in the T21 pregnancy group than in euploid pregnancies. Furthermore, the screening utility was proved by AUC = 0.978, with the cut-off point set at 1.37 mg/mL. This T21 screening marker was characterized by 80% sensitivity and 100% specificity [9].

Following the evaluation of disturbed metabolic pathways in T21 pregnancies, where the lipid pathway is inherently connected to the carbohydrate pathway, the novel insulin-resistance marker protein asprosin and advanced glycation end products (AGEs) were evaluated for T21 prenatal screening. The beneficial role of asprosin measurement in prenatal screening was characterized by 100% sensitivity, 85% specificity, and AUC = 0.965. The AGE assessment showed 80% specificity and 81% sensitivity for screening. Furthermore, the SOD-2 genes mapped on chromosome 21 and the impact of oxidative stress on T21 development have been studied [95,96,97,98,99]. Accordingly, oxidative stress markers were evaluated for T21 prenatal screening. It was proved that measuring the products of DNA/RNA damage induced by oxidative stress could be a novel tool in T21 prenatal screening. Moreover, the anti-inflammatory protein α-1-antitrypsin (A1AT), which has antioxidative properties, was also suggested as a novel T21 screening marker [100]. Interestingly, the level of A1AT was found to be downregulated in T21 aneuploidy. The results suggest that the decrease in A1AT concentration combined with aggravated inflammation processes and oxidative stress observed in T21 pregnancies may negatively impact multiple comorbidities and the occurrence of fetal malformations. The proposed novel T21 screening markers are characterized in Table 4 [101].

## 8. Discussion

The current trend in prenatal testing represents a massive rearrangement from invasive to non-invasive or less-invasive sampling procedures [102]. The introduction of noninvasive prenatal testing using cffDNA was a breakthrough moment for prenatal aneuploidy screening [103]. It is reasonable to anticipate that further advances in prenatal screening development could lead to improvements in biochemical screening accuracy, following the promising result obtained by the potential introduction of omics methods into the prenatal screening [104]. Our review reveals that, while the successful use of omics techniques in prenatal screening has been reported, many challenges still exist. Moreover, the omics methods can be used for rapid and precise screening of large amounts of samples. Unfortunately, processing of biological material often requires complex preparations, a large amount of various reagents, and the work of a specialized group of scientists [105]. Moreover, the testing remains expensive, which limits its introduction into routine diagnostics, especially in less-developed and medium-developed countries [106]. From the other hand, the complexity of omics data analysis requires data integration and pipeline validation supported by bioinformatics and biostatistics.

Therefore, constantly updated databases are needed to standardize the proposed T21 biomarker reference values and improve data management. Further integration of these approaches exploits the advantages of these techniques, providing a rapid and accurate on-site method for T21 detection and quality control of potential screening biomarker validation [107]. In this case, omics methods could be effectively incorporated from research laboratories to everyday routine diagnostics as costs and processing time for sample analyses continue to decrease, which has been noticed. This translates to an increased contribution of omics methods in clinical trials, which may result in including them in the standards and recommendations of diagnostic procedures [103,108]. Especially in prenatal screening—the results obtained with the used of omics could be particularly useful in the early and precise fetal defects screening. Up to date, ELISA was a useful tool which meet the challenge of introducing results obtained with omics-based methods into daily routine diagnostics with subsequent validation of different procedures. Accordingly, due to the recent advance in genomics, current prenatal testing has evolved mainly to cell-based assays and cffDNA [103].

However, there is still a need for novel, omics-based studies in order to improve T21 prenatal screening and, more importantly, to discover potential medical targets. Proposed T21 therapy has focused on pharmacological treatment to improve cognition. A number of compounds have been shown to exhibit potential beneficial properties, reported to improve learning and congenital anomalies [109]. Chronic treatment with picrotoxin or pentylenetetrazol improved deficits in hippocampus-based learning and long-term potentiation. Nevertheless, these trials are still carried out on a mouse model, Ts65Dn (which displays various DS phenotypes), which extends the time before the proposed solutions are implemented in routine clinical management [20,21,22]. The integrated use of omics in T21 evaluation should be thoroughly investigated in the nearly future [110]. Personalized medicine and omics technologies together provide global understanding of the mechanisms responsible for T21 occurrence. Advances in omics results should be correlated with the congenital disabilities and others comorbidities occurring during T21 development, moving this trend toward a personalized medicine and management course to clarify the molecular mechanisms underlying T21 pathogenesis. Simultaneously, the discovery of potential prenatal biomarkers and therapeutic targets could provide more detailed patient stratification and personalized treatment improving clinical management [111].

## 9. Conclusions

The introduction of integrated omics methods into routine non-invasive prenatal screening could increase the detection rate of fetal aneuploidy including T21. Based on our literature search, it can be concluded that cbDNA and cffDNA analysis demonstrate the vast potential in NIPT. However, there is still a need to provide useful data in order to validate their usefulness. Moreover, the development of fully automated systems remains essential to introduce modern technologies in prenatal screening. Accordingly, novel approaches have provided new insights into the complex pathophysiology of T21, which could be further used in novel therapeutic strategy evaluation.

## Figures and Tables

**Table 1:** Biochemical prenatal screening markers [10,20,21,22,23,24].

Pregnancy Period	Ultrasound	Biochemical Test	Sensitivity	Specificity
First trimester (11–13 weeks)	+ (NT)	PAPP-A and free βhCG	85–90%	82–87%
Second trimester (18–24 weeks)	+	βhCG + uE3 + AFP + inhibin A	69–92%	81–96%
First or second trimester	+	PAPP-A, AFP, uE3, total hCG	88%	90–95%
First or second trimester	+	PAPP-A, inhibin A, AFP, uE3, free βhCG/total hCG	85%	90–95%

AFP, alpha-fetoprotein; βhCG, chorionic gonadotropin beta subunit; hCG, chorionic gonadotropin; uE3, unconjugated estradiol; NT, nuchal translucency; PAPP-A, pregnancy-associated plasma protein A.

**Table 2:** Comprehensive list of discriminating metabolites that can serve as reliable biomarkers in T21 prenatal screening (*p* < 0.05) [58,63].

Biological Sample	Significant Deregulated Metabolites in T21 Prenatal Screening
maternal blood	2-hydroxybutyrate, alanine, citric acid, phenylalanine, 3-methyl histidine, proline, benzoic acid, glyceric acid, mannose, myristic acid, stearic acid
maternal serum	2-hydroxybutyrate, 3- hydroxybutyrate, acetone, glycerol, glycine, isobutyrate, ornithine, phenylalanine, succinate, methylhistidine, arginine, 12-hydroxybutyrate, carnitine, lactate, pyruvate, dimethylamine, methionine
maternal plasma	butyryl-l-carnitine, palmitic amide, linoleamide, oleamide, piperine, proline, methanol, creatinine
maternal urine	dihydrouracil, methanol, β-hydroxybutyrate
amniotic fluid	methylhistidine, hexanoylcarnitine, diacetylspermine, and p-cresol sulfate

**Table 3:** Utility of T21 prenatal screening panels.

T21 Screening Panel	AUC Based on ROC Curves
βhCG + PAPP-A	0.918
PAPP-A + NT	0.922
PAPP-A + hCG-sLHCGR	0.920
βhCG + hCG-sLHCGR	0.856
βhCG + NT	0.753
hCG-sLHCGR + NT	0.888
NT + PAPP-A + βhCG	0.940
NT + PAPP-A + hCG-sLHCGR	0.928
hCGsLHCGR + NT + PAPP-A + βhCG	0.966

AUC, area under the receiver operating characteristic (ROC) curve; βhCG, chorionic gonadotropin beta subunit; hCG-sLHCGR, human chorionic gonadotropin with its cognate receptor LH/hCG-R or LHCGR; NT, nuchal translucency; PAPP-A, pregnancy-associated plasma protein A.

**Table 4:** Diagnostic utility of tested novel screening markers.

Marker	Unit	AUC	Cut-Off Value	Sensitivity	Specificity
ApoE	ng/mL	0.978	>1.37	80%	100%
Asprosin PS	ng/mL	0.970	>12.70	100%	85%
Asprosin AF	ng/mL	0.830	>12.91	95%	65%
AGE PS	ng/mL	0.850	<11.00	81%	80%
AGE AF	ng/mL	0.960	<4.184	95%	90%
A1AT PS	mg/L	0.530	<2.341	81%	33%
A1AT AF	mg/L	0.870	<0.3180	76%	86%
DNA/RNA OSDP PS	pg/mL	0.510	<40.30	80%	40%
DNA/RNA OSDP AF	pg/mL	0.730	>31.76	84%	58%

A1AT, alpha-1-antitrypsin; AF, amniotic fluid; ApoE, apolipoprotein E; AGE, advanced glycation end product; AUC, area under receiver operating characteristic (ROC) curve; OSDP, oxidative stress damage product; PS, plasma.

## Data Availability

Not applicable.
